# Cell biology and the curse of carbon

**DOI:** 10.1007/s00709-022-01827-1

**Published:** 2022-12-03

**Authors:** Peter Nick

**Affiliations:** Botanical Institute, Karlsruhe Institute of Technology, Karlsruhe, Germany

Global food security has shifted into public attention — a few days ago, at the COP27 summit in Sharm-el Sheikh, the impact of climate change upon agricultural production had been a central issue, and the shortage of wheat in consequence of the Russian invasion into the Ukraine has demonstrated the vulnerability of supply, leading to sharp increases of bread prices and social tensions in many countries. A recent study shows that, already now, the negative impact of climate change overruns the breeding success on wheat yield in North America (Zhang et al. 2022). However, endangered food security is not the only negative consequence of non-sustainable use of fossil fuels. Already their exploitation comes with a lot of collateral damage to the environment, such as pollution of the most precious resource we all rely upon — water. Thus, the curse of fossil carbon can be felt throughout its life cycle — from the very moment that it is extracted from the ground till the moment that its remnants heat up our atmosphere. Three contributions to the current issue, all from countries existentially challenged by this price for carbon economy, highlight how cell biology can help to address these issues.

The contribution by Mohi-ud-Din et al. (2023) deals with heat tolerance in wheat. Every °C of mean temperature means a yield loss of 6% (Asseng et al. 2015), due to a pronounced temperature sensitivity during flower development and seed filling. In other words, if the annual temperatures rise, by a joint effort of all societies on our planet, not beyond the currently ventilated 1.5 °C, wheat yield is projected to drop by 9%. While this might seem an acceptable loss, one needs to keep in mind that the average temperature increase reflects the real challenge only partially because it is also the timing of heat stress episodes that matters. For instance, in the Indian Ganges plain as well as in Bangladesh (where the authors originate from), wheat is grown in rotation with rice, and since the monsoon season is delayed progressively, sowing of wheat is shifted in time, such that the probability of experiencing a stress episode upon anthesis or during seed filling increases much more than one would infer from the rise in average temperature (Dubey et al. 2020). Even a relatively mild increase by 5 °C in the sensitive period is sufficient to cause a significant drop in grain yield. Usually, a mild increase in temperature is expected to accelerate biochemical processes at a Q_10_ of 3 (meaning that rising the temperature by 10 °C should speed up biochemistry by a factor of three). From a naive viewpoint, mild temperature increases should boost growth rather than cause damage. However, there are very sensitive processes that suffer significantly when kinetic homeostasis is perturbed. One of these processes is photosynthetic electron transport across the thylakoid membrane. When more electrons are generated than can be consumed by the formation of reduction equivalents, they can easily end up at the molecular oxygen resulting from water splitting in photosystem II. As a result, reactive oxygen species accumulate which can damage not only membranes but also proteins and nucleic acids. The authors focus, therefore, not only on the redox homeostasis and monitoring of lipid peroxidation (formation of malone dialdehyde as readout) but also enzymatic antioxidant systems, such as the ascorbate and glutathione cycle, as well as glyoxylate detoxification (Allan et al*.* 2009). By introgression of a chromosome segment (2NvS) from the wild wheat relative *Aegilops ventricosa* (Gao et al. 2021) into bread wheat, the authors have generated two lines with good yield and high thermotolerance. They study their performance under real-world conditions by cultivating them at the standard season or under delayed sowing, as it is getting a new practice due to the delayed rice harvest. They can confirm that the introgression lines perform much better compared to the reference line Pavon — under heat stress one of the lines produces almost twice the yield as seen in Pavon. The introgressed chromosome segment is densely populated with genes for cytochrome P450 enzymes involved in secondary metabolism and detoxification, some of which have already been shown to be upregulated by heat stress. Overall, this contribution shows how the use of resilience genes from crop wild relatives in combination with analysis of cellular mechanisms can help to breed novel crop plants that are able to yield under stress.

In their contribution, Tounsi et al. (2023) focus on one of the central players of redox homeostasis, superoxide dismutases (SODs), enzymes that can convert superoxide, one of the most dangerous reactive oxygen species, into hydrogen peroxide, which can then be further detoxified by catalase. The SODs are encoded by a gene family with individual members acting in different subcellular locations. The mitochondrial and plastidic SODs dissipate the superoxide, which is generated as byproduct upon perturbation of electron transport due to stress. In addition, cytoplasmic SODs kick in, when this organelle response is not sufficient, such that reactive oxygen species leak out, and apoplastic SODs play a role in signalling and in the interaction with pathogens. The study centres on Durum Wheat, a drought-tolerant wheat species that derives from *Emmer* Wheat, the tetraploid ancestor of modern bread wheat. Durum wheat was developed twice, in the Near East and, probably independently, in Ethiopia (Tidiane Sall et al. 2019) and is widespread in the not only in the Mediterranean and in North and East Africa but also in other arid regions of the world. Durum is often used for iconic regional foods such as pasta or couscous and, thus, it is important for regional identities. In short, Durum has the potential to turn into something like a “crop of climate change”. However, unlike its offspring, the hexaploid bread wheat, the molecular details of durum wheat have not been studied with the same intensity. The authors have, therefore, mined the durum genome for the SOD family which is found in a total of 14 homologues that cluster into three families. The analysis of promoter regions reveals numerous motifs needed for abiotic stress regulation, and the authors follow the expression of eight candidates in response to drought and cold, as well as treatment with ABA, a phytohormone accumulating under drought. They also address salinity, a pertinent issue in Tunisia, where these authors come from. The study shows qualitative differences in regulation — some SODs are responsive to all stress types; others, such as SOD7B and SOD2B only to cold stress, while SOD2B-2 is upregulated in response to ABA. The identification of SOD isoforms that are specifically regulated along with the knowledge of the details in their promoter region provide valuable tools for marker-assistant breeding, where durum landraces can be screened for the presence of a candidate promoter signature and then introgressed into high-yielding durum varieties to breed a resilient wheat that will provide food security even under the challenge of climate change.

While the two contributions above develop solutions to deal with the consequences of fossil fuel, the contribution by Alabi (2023) scrutinises on the upstream steps of a fossil economy, addressing the collateral damage of petroleum refining, an important industrial activity in Nigeria, where the author is living. He uses a combination of cellular assays and chemical analytics to assess the ecological impact of wastewater from a petroleum refinery on the water quality in the receiving river. A panel of bacterial assays demonstrate that the wastewater exerts an unacceptable degree of mutagenicity; the formation of micronuclei in a fish cell line gives evidence that there is genotoxicity as well. The author also demonstrates that the bacterial strains differ with respect to genotoxic sensitivity. The data with bacteria and cell lines are complemented by a comprehensive study with catfishes in response to water from the refinery and the receiving river that shows a significant decrease of blood quality parameters. The chemical analysis detects elevated levels of toxic compounds, such as benzene, toluene, or polycyclic aromatic hydrocarbons that derive from petroleum refining. However, also heavy metals such as cadmium, mercury, nickel, lead, and vanadium can be found. This study illustrates how environmental pollution can be traced down at low cost using cell biological assays. It also highlights how fossil fuel, from the very beginning, charges high costs to health and the environment. The author concludes his work with a plea to the Nigerian government to hold negligent refineries accountable to deter future contamination.

All three contributions use cell biology to address existential challenges of the real world. Whether heat resilience of bread wheat is promoted by introgression of a chromosomal fragment from a robust crop wild relative (Mohi-ud-Din et al. 2023), whether a key factor of redox homeostasis in the drought tolerant ancient crop durum wheat is systematically analysed to find new genetic factors for resilience breeding (Tounsi et al. 2023), or whether cellular assays are used to identify sources of pollution (Alabi 2023) — all these examples show very clearly that science has a real impact to improve and safeguard the lives of many people.
